# Impact of Bicarbonate on PBP2a Production, Maturation, and Functionality in Methicillin-Resistant Staphylococcus aureus

**DOI:** 10.1128/AAC.02621-20

**Published:** 2021-04-19

**Authors:** Selvi C. Ersoy, Henry F. Chambers, Richard A. Proctor, Adriana E. Rosato, Nagendra N. Mishra, Yan Q. Xiong, Arnold S. Bayer

**Affiliations:** a The Lundquist Institute, Torrance, California, USA; b UCSF School of Medicine, San Francisco, California, USA; c Departments of Medicine and Medical Microbiology/Immunology, University of Wisconsin School of Medicine and Public Health, Madison, Wisconsin, USA; d Department of Pathology, Riverside University Health Systems, Riverside, California, USA; e Geffen School of Medicine at UCLA, Los Angeles, California, USA

**Keywords:** methicillin-resistant *Staphylococcus aureus* (MRSA), sodium bicarbonate (NaHCO_3_), beta-lactam, penicillin-binding proteins (PBP), PBP2a

## Abstract

Certain methicillin-resistant Staphylococcus aureus (MRSA) strains exhibit β-lactam susceptibility *in vitro*, *ex vivo*, and *in vivo* in the presence of NaHCO_3_ (NaHCO_3_-responsive MRSA). Here, we investigate the impact of NaHCO_3_ on factors required for PBP2a functionality. Prototype NaHCO_3_-responsive and -nonresponsive MRSA strains (as defined *in vitro*) were assessed for the impact of NaHCO_3_ on the expression of genes involved in PBP2a production-maturation pathways (*mecA*, *blaZ*, *pbp4*, *vraSR*, *prsA*, *sigB*, and *floA*), membrane PBP2a and PrsA protein content, and membrane carotenoid content. Following NaHCO_3_ exposure in NaHCO_3_-responsive (versus nonresponsive) MRSA, there was significantly reduced expression of (i) *mecA* and *blaZ*, (ii) the *vraSR-prsA* gene axis, and (iii) *pbp4*. Carotenoid production was reduced while *floA* expression was increased by NaHCO_3_ exposure in all MRSA strains. This work underscores the distinct regulatory impact of NaHCO_3_ on a cadre of genes encoding factors required for the maintenance of the MRSA phenotype through PBP2a functionality and maturation.

## Abstract (precis)

Certain methicillin-resistant Staphylococcus aureus (MRSA) strains exhibit β-lactam susceptibility *in vitro*, *ex vivo*, and *in vivo* in the presence of NaHCO_3_ (NaHCO_3_-responsive MRSA). Here, we investigate the impact of NaHCO_3_ on factors required for PBP2a functionality.

## INTRODUCTION

Staphylococcus aureus is the major causative agent of a number of serious clinical syndromes and a notable public health threat (1–3). Methicillin-resistant S. aureus (MRSA) has been a particular problem due, in part, to its assumed recalcitrance to many first-line therapies useful in the treatment of methicillin-susceptible S. aureus (MSSA) strains, especially β-lactam agents (4). Current anti-MRSA therapies tend to be costlier, less effective, and/or more toxic than those used to treat MSSA infections (e.g., daptomycin, linezolid, and vancomycin [5, 6]); also, patients with MRSA infections are prone to prolonged hospital stays, imposing large economic burdens for their treatment (7).

MRSA strains are preemptively identified in most clinical microbiology laboratories by phenotypic assays (e.g., cefoxitin disk diffusion or PBP2a latex agglutination) (8), as well as by the presence of the *mecA* gene cassette, encoding the alternative penicillin-binding protein 2a (PBP2a). Additionally, minimum inhibitory concentration (MIC) determinations, as outlined by the Clinical and Laboratory Standards Institute (CLSI), are used to confirm their resistance to standard β-lactam antibiotics (i.e., oxacillin) (9, 10). The CLSI considers S. aureus isolates whose MICs to oxacillin are ≥4 μg/ml in cation-adjusted Mueller-Hinton broth (CA-MHB) to be oxacillin resistant (and resistant to first-generation cephalosporins) (4, 9); the treatment of such strains with all β-lactam agents (excluding ceftaroline and ceftobiprole) is discouraged by published guidelines (4, 9).

We recently discovered that the addition of NaHCO_3_, the body’s primary biological buffer, to CA-MHB rendered a relatively large proportion of MRSA strains susceptible to two standard β-lactams, oxacillin and cefazolin, by MIC testing; this phenotype has been termed NaHCO_3_ responsiveness (11, 12). The translational relevance of this NaHCO_3_-responsive phenotype was verified in a small strain set by successful β-lactam therapy in an *ex vivo* simulated endocarditis vegetation model as well as in a rabbit model of infective endocarditis (11, 13).

Prior studies suggested that NaHCO_3_ exerted its impact on antimicrobial susceptibility by targeting and collapsing the proton motive force (PMF) (14); however, this would not adequately explain β-lactam resensitization by NaHCO_3_. Instead, we recently determined that coexposure of these *in vitro*-responsive MRSA strains to NaHCO_3_ plus oxacillin reduced the expression of both *mecA* and *sarA*, associated with reduced PBP2a protein production (11). The latter observations were important, as oxacillin binds relatively poorly to PBP2a (encoded by *mecA*), and deletion of *sarA* can increase susceptibility to oxacillin, in part, by influencing *mecA* expression (15).

The above-described finding of NaHCO_3_-mediated reduction of *sarA* expression suggested an additional mechanism by which NaHCO_3_ could influence β-lactam sensitization in MRSA, i.e., via an impact on cell membrane (CM) physiology and PBP2a maturation. The expression of *sarA* is regulated by the alternative sigma factor, SigB (16), which, in turn, controls the staphyloxanthin biosynthesis operon, *crtOPQMN* (17). The staphyloxanthin carotenoid pigment, along with flotillin (encoded by *floA*), are integral parts of functional membrane microdomains (FMMs), which, importantly, provide a scaffolding that anchors PBP2/2a proteins within the MRSA CM (18, 19).

Defining the potential impacts of NaHCO_3_ on other key aspects of PBP2a regulation (e.g., *blaZ* expression), maturation (e.g., *vraSR* and *prsA* expression), and peptidoglycan cross-linking (e.g., *pbp4*) is critical to developing a more complete understanding of bicarbonate’s effect on oxacillin susceptibility. Thus, genes within the *blaZ* operon coregulate *mecA* expression (20). Of note, VraSR and PrsA have been identified as critical to the see-saw effect, in which daptomycin-nonsusceptible MRSA strains exhibit β-lactam hypersusceptibility (21–23). VraSR is a two-component regulatory system that positively regulates the expression of PrsA, a chaperone required for proper PBP2a maturation, localization, and functionality (24, 25). Moreover, PBP4 is a PBP with transpeptidase activity required to complete peptidoglycan cross-linking in the presence of oxacillin (26, 27).

The current study was designed to adjudicate the above-described preliminary findings in a larger collection of NaHCO_3_-responsive and -nonresponsive MRSA strains as well as to further define key genetic determinants of these two distinct microbiologic phenotypes.

(These data were presented, in part, at the 4th Annual Texas Medical Center Antimicrobial Resistance and Stewardship Conference [28].)

## RESULTS

### Impact of NaHCO_3_ on *mecA* and *blaZ* expression.

**FIG 1:**
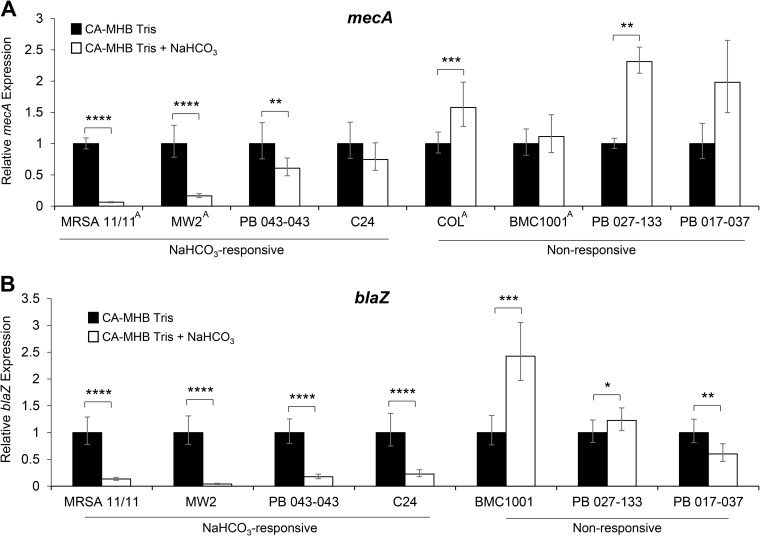
Expression of *mecA* (A) and *blaZ* (B) in the presence and absence of NaHCO_3_. qRT-PCR was performed using RNA extracted from stationary-phase cells in the presence of 0.5× MIC of oxacillin. Oxacillin concentrations used are the following: MRSA 11/11, without NaHCO_3_ (w/o), 16 μg/ml; with NaHCO_3_ (+ NaHCO_3_), 0.25 μg/ml; MW2, w/o, 32 μg/ml; + NaHCO_3_, 1 μg/ml; PB 043-043, w/o, 8 μg/ml; + NaHCO_3_, 0.5 μg/ml; C24, w/o, 16 μg/ml; + NaHCO_3_, 0.5 μg/ml; COL, w/o, 256 μg/ml; + NaHCO_3_, 256 μg/ml; BMC1001, w/o, 128 μg/ml; + NaHCO_3_, 128 μg/ml; PB 027-133, w/o, 128 μg/ml; + NaHCO_3_, 128 μg/ml; PB 017-037, w/o, 16 μg/ml; + NaHCO_3_, 16 μg/ml. Black bars indicate growth in CA-MHB 100 mM Tris, white bars indicate growth in CA-MHB 100 mM Tris plus 44 mM NaHCO_3_, and error bars indicate the *C_T_* standard deviations. No *blaZ* expression was detected in COL, because the strain lacks the *blaZ* gene. Student’s *t* test was used for statistical analyses. *, *P* < 0.05; **, *P* < 0.01; ***, *P* < 0.001; ****, *P* < 0.0001. *mecA* gene expression data for strains marked with a superscript A were previously published (11).

The *blaZ* operon is important in coregulating *mecA* expression and PBP2a production (20). As shown in Fig. 1, NaHCO_3_ exposure substantially decreased *mecA* gene expression under oxacillin-inducing conditions in all four NaHCO_3_-responsive strains. In contrast, *mecA* expression was increased during NaHCO_3_ exposure in the four nonresponsive strains (Fig. 1A). Similarly, *blaZ* expression was significantly reduced in all four NaHCO_3_-responsive strains while being significantly increased in two of the three β-lactamase-positive, NaHCO_3_-nonresponsive strains (COL is naturally β-lactamase negative) (Fig. 1B).

### Influence of NaHCO_3_ on PBP2a content.

As previously observed in a smaller set of MRSA strains (11), growth in media containing NaHCO_3_ reduced the amount of PBP2a in NaHCO_3_-responsive versus nonresponsive strains (Table 1; see also Fig. S2 in the supplemental material).

**TABLE 1:** PBP2a agglutination of NaHCO_3_-responsive and nonresponsive strains grown in medium with or without 44 mM NaHCO_3_

PBP2a agglutination level^*a*^	
Strain	CA-MHB Tris	CA-MHB Tris 44 mM NaHCO_3_
MRSA 11/11^*b*^	++	−
MW2^*b*^	++	−
PB 043-043	+++	+
C24	+++	+
COL^*b*^	+++	+++
BMC1001^*b*^	+++	+++
PB 027-133	+++	+++
PB 017-037	+++	+++

a Interpretation of agglutination intensity: +++, high; ++, moderate; +, low; −, none.

b Data were previously reported (11).

**FIG 2:**
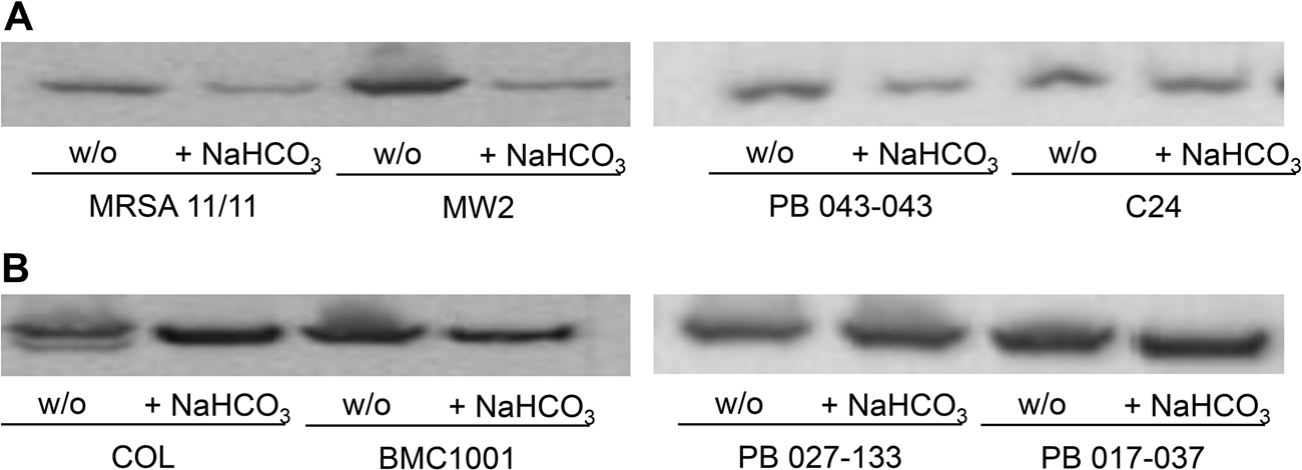
Membrane PBP2a protein content in MRSA strains grown in the presence and absence of NaHCO_3_. (A) NaHCO_3_-responsive strains. (B) NaHCO_3_-nonresponsive strains. Strains were grown in CA-MHB plus 100 mM Tris (w/o) or CA-MHB plus 100 mM Tris plus 44 mM NaHCO_3_ (+ NaHCO_3_) in the presence of 0.5× MIC oxacillin. Proteins are the cell membrane fraction extracted from stationary-phase cells.

To further verify the impact of NaHCO_3_ on PBP2a protein production and its CM localization, Western blotting was performed on CM protein extracts from cells grown in the presence of 0.5× MIC of oxacillin (to induce *mecA* expression), with or without NaHCO_3_ (Fig. 2). As predicted by the known NaHCO_3_ repression of the coregulatory *mecA-blaZ* gene axis expression in NaHCO_3_-responsive strains, CM-specific PBP2a protein production was visibly decreased in the presence of NaHCO_3_ in three of four NaHCO_3_-responsive strains (Fig. 2A). In contrast, NaHCO_3_ exposure had no impact on PBP2a production in any of the NaHCO_3_-nonresponsive strains (Fig. 2B). These data indicated that not only was total PBP2a production downregulated in NaHCO_3_-responsive strains (as per the agglutination assay) but also the amount of protein specifically inserted into the CM was similarly impacted.

### Effect of NaHCO_3_ on genes involved in PBP2a maturation and localization.

**FIG 3:**
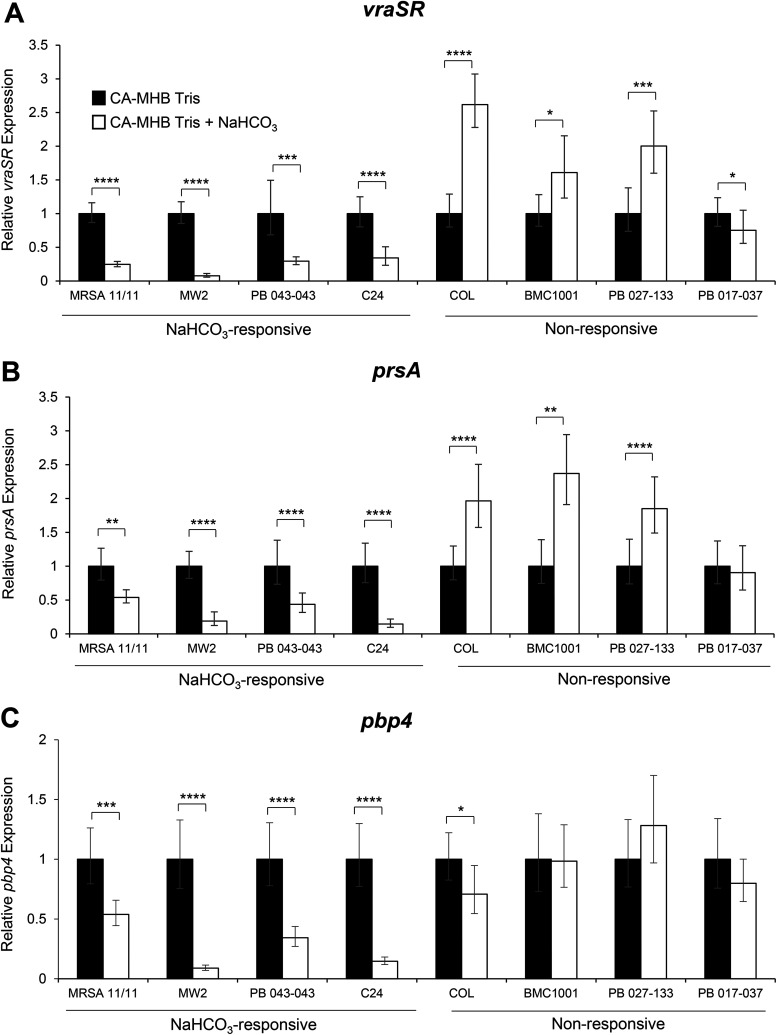
Expression of genes related to PBP2a maturation and function in the presence and absence of NaHCO_3_. (A) *vraSR.* (B) *prsA.* (C) *pbp4*. qRT-PCR was performed using RNA extracted from log-phase cells (OD_600_, 0.4) in the presence of 0.5× MIC oxacillin for *vraSR* and *prsA* or on RNA extracted from stationary-phase cells (*pbp4*). Black bars indicate growth in CA-MHB with 100 mM Tris, white bars indicate growth in CA-MHB with 100 mM Tris plus 44 mM NaHCO_3_ for all graphs, and error bars indicate the *C_T_* standard deviations. Student’s *t* test was used for statistical analyses. *, *P* < 0.05; **, *P* < 0.01; ***, *P* < 0.001; ****, *P* < 0.0001.

For optimal PBP2a protein functionality, the PrsA chaperone must facilitate posttranslational maturation of PBP2a (21, 25). Regulation of *prsA* gene expression typically occurs via VraSR, a two-component regulatory system known to be involved in the daptomycin resistance/oxacillin hypersusceptibility see-saw effect (21, 23). Interestingly, NaHCO_3_ highly repressed expression of both *vraSR* and *prsA* only in NaHCO_3_-responsive strains (Fig. 3A and B).

**FIG 4:**
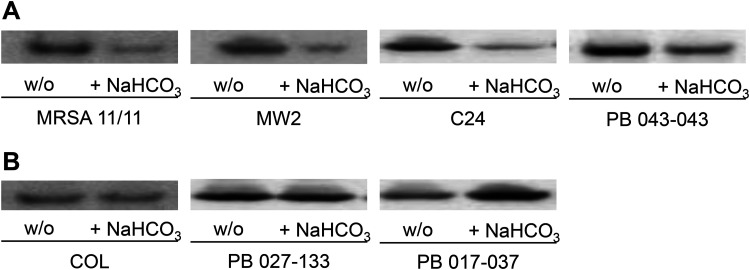
Membrane PrsA protein content in MRSA strains grown in the presence and absence of NaHCO_3_. (A) NaHCO_3_-responsive strains. (B) Nonresponsive strains. Strains were grown in CA-MHB plus 100 mM Tris (w/o) or CA-MHB plus 100 mM Tris plus 44 mM NaHCO_3_ (+ NaHCO_3_) in the presence of 0.5× MIC oxacillin. Proteins are the membrane fraction extracted from log-phase cells.

Furthermore, as predicted by the quantitative real-time PCR (qRT-PCR) gene expression data described above, less PrsA protein was present in the CMs of all NaHCO_3_-responsive MRSA following NaHCO_3_ exposure versus cells grown in the absence of NaHCO_3_ (Fig. 4A). In contrast, NaHCO_3_ had no apparent effect on PrsA CM content in three NaHCO_3_-nonresponsive strains (Fig. 4B). NaHCO_3_ did not consistently impact extracellular secretion patterns of PrsA in either NaHCO_3_-responsive or -nonresponsive MRSA (data not shown). These data support the notion that in the presence of oxacillin induction, NaHCO_3_ reduces overall PrsA production in NaHCO_3_-responsive MRSA, resulting in decreased PrsA CM content (as opposed to excess PrsA secretion extracellularly, as seen in the see-saw effect [21]).

### *pbp4* expression.

In addition to proper protein folding, the PBP2a protein requires PBP4 activity to complete cell wall synthesis (26). Although PBP2a provides the transpeptidase activity involved in peptidoglycan synthesis, highly cross-linked peptidoglycan cannot be produced in many MRSA strains in the presence of oxacillin without PBP4 (18, 26, 29). The qRT-PCR analysis revealed that *pbp4* expression was substantially and selectively repressed in the presence of NaHCO_3_ in NaHCO_3_-responsive (but not in NaHCO_3_-nonresponsive) strains (Fig. 3C); this suggested that a less highly cross-linked peptidoglycan is produced in NaHCO_3_-responsive strains in the presence of NaHCO_3_.

### Influence of NaHCO_3_ on factors impacting FMM scaffolding for PBP2a.

The alternative sigma factor and stress response regulator, SigB, is involved in regulating genes involved in CM biophysics (e.g., fluidity/rigidity), notably the staphyloxanthin carotenoid biosynthesis operon, *crtOPQMN* (17). In turn, carotenoids and flotillin (FloA) are critical in providing a key scaffolding upon which PBP2a can oligomerize (19). Additionally, the global regulon *sarA* is also SigB regulated and is important in maintaining the MRSA phenotype (15, 16, 30). Further, as noted above, we previously determined that NaHCO_3_ was capable of repressing *sarA* expression in NaHCO_3_-responsive strains (11). Thus, we hypothesized that NaHCO_3_ influences SigB activity, resulting in downstream impacts on SigB-regulated phenotypes important in PBP2a functionality.

**FIG 5:**
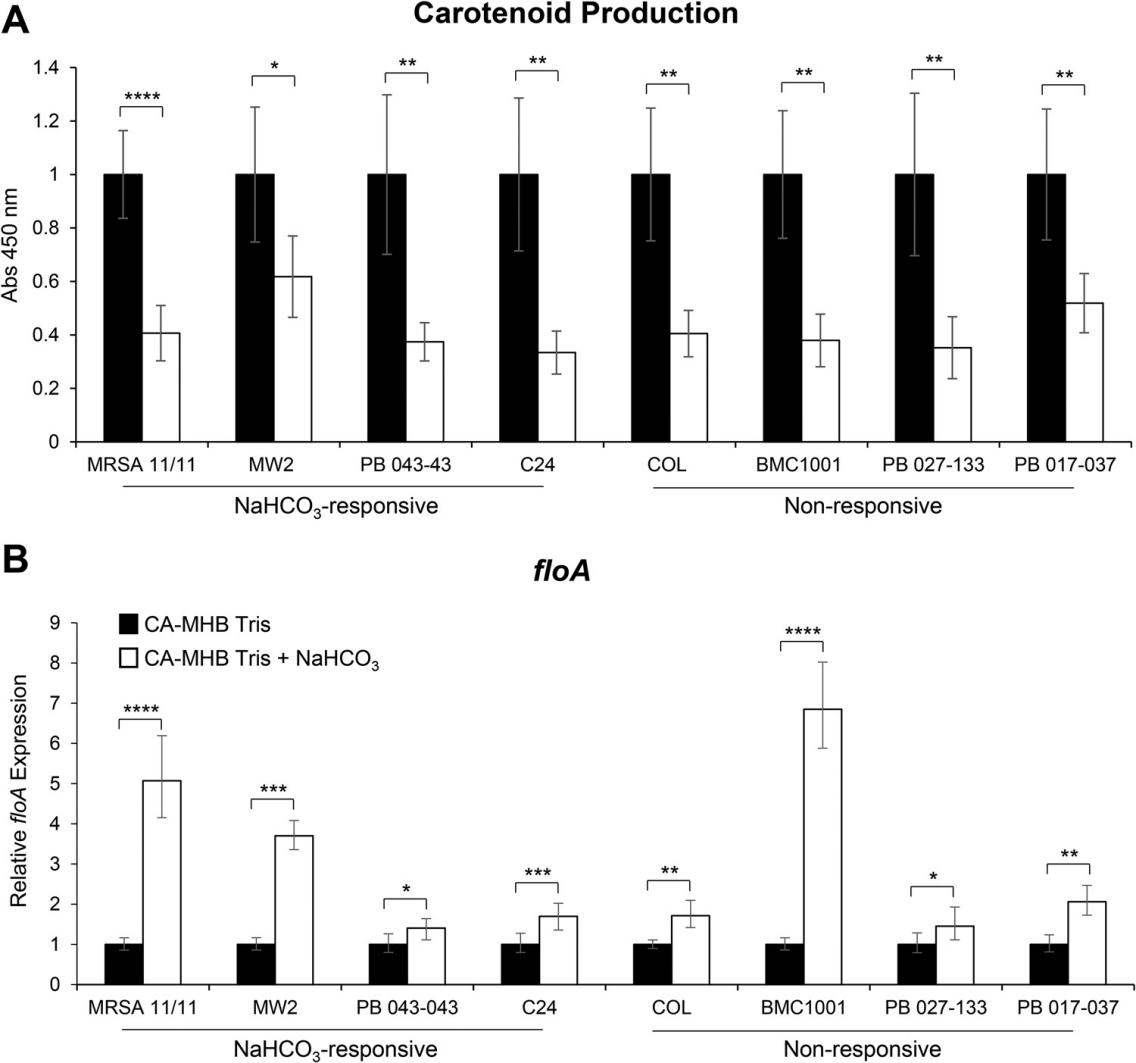
Impact of NaHCO_3_ on FMM-associated factors. (A and B) Carotenoid production (A) and *floA* gene expression (B) in MRSA strains grown in the presence and absence of NaHCO_3_. *floA* gene expression was assessed by qRT-PCR using RNA extracted from log-phase cells (OD_600_, 0.4) in the presence of 0.5× MIC oxacillin. Black bars indicate growth in CA-MHB with 100 mM Tris, white bars indicate growth in CA-MHB with 100 mM Tris plus 44 mM NaHCO_3_ for all graphs, and error bars indicate the *C_T_* standard deviations. Student’s *t* test was used for statistical analyses. *, *P* < 0.05; **, *P* < 0.01; ***, *P* < 0.001; ****, *P* < 0.0001.

The expression of the constitutively active *sigB* gene was assessed through expression of *asp23*, a surrogate reporter for *sigB* promoter activity (31). Interestingly, in both NaHCO_3_-responsive and -nonresponsive strains, *asp23* expression was substantially and uniformly repressed in the presence of NaHCO_3_ (data not shown). To confirm the phenotypic outcome of such globally repressed SigB activity, carotenoid content was measured in NaHCO_3_-responsive and -nonresponsive strains and was found to be universally reduced by NaHCO_3_ exposures (Fig. 5A).

Since carotenoids interact with flotillin within the CM to form FMMs (19), we determined whether NaHCO_3_ also impacts the flotillin component of FMMs by assessing the expression of *floA*. Interestingly, *floA* gene expression was increased in all strains in the presence of NaHCO_3_ (Fig. 5B). Combined with the reduced carotenoid content data described above, the latter data indicate that NaHCO_3_ is inducing a proportionality disequilibrium in the formation of stable FMMs (i.e, reduced carotenoids plus increased *floA* expression).

## DISCUSSION

Previously, we identified a novel MRSA phenomenon, termed NaHCO_3_ responsiveness, wherein a relatively large proportion of MRSA strains displayed increased susceptibility to standard β-lactam antibiotics in the presence of NaHCO_3_ (11, 12). Thus, ∼75% and ∼33% of a collection of 58 recent MRSA bloodstream isolates were significantly more susceptible *in vitro* to cefazolin and oxacillin, respectively, in the presence versus absence of NaHCO_3_ (12). We also linked this responsiveness phenomenon to the reduced expression of two genes involved in the maintenance of the MRSA phenotype, *mecA* and *sarA* (11). These genetic impacts translated into reduced PBP2a production in such NaHCO_3_-responsive MRSA in the presence of NaHCO_3_.

In the present study, we further investigated additional mechanisms potentially involved in NaHCO_3_–β-lactam responsiveness, in eight representative strains, focusing on the impact of NaHCO_3_ upon both *mecA*-*blaZ* coregulation and key genes involved in the maturation functionality of PBP2a; each of these events is critical in determining ultimate MRSA β-lactam resistance.

Several interesting observations emerged from these investigations. First, in the presence of oxacillin induction, NaHCO_3_ substantially reduced the expression of both *mecA* and *blaZ* only in NaHCO_3_-responsive strains. Thus, it seems clear that in NaHCO_3_-responsive strains, NaHCO_3_ impacts both limbs of the *mecA-blaZ* coregulator*y* axis important in the maintenance of the MRSA phenotype (20). Also, our prior studies, utilizing a latex agglutination assay, showed that NaHCO_3_ could reduce overall production of PBP2a in NaHCO_3_-responsive versus -nonresponsive MRSA (11). Similar PBP2a production differences were confirmed using this same assay in additional strains as well as with a more precise Western blot assay for CM localization of this PBP.

Second, we established that NaHCO_3_ exposure *in vitro* had a rather profound dampening effect on the expression of several key genes involved in PBP2a maturation. For example, the two-component regulatory system, VraSR, and the VraSR-regulated chaperone, PrsA, are known to work in concert; dysfunction in this circuit appears to underlie the see-saw effect, wherein daptomycin-resistant MRSA strains become hypersusceptible to β-lactams (21, 22). Interestingly, such see-saw strains actually demonstrate increased expression of both *vraSR* and *prsA* as well as the accumulation of mutations in *mprF* (21, 23). The collateral effects of the latter genetic changes are the decreased production of PrsA in the CM and concomitant increased extracellular secretion of PrsA, with a resultant decreased localization of PBP2a in the CM (21). In contrast, in NaHCO_3_-responsive MRSA, there appears to be a totally different mechanism involved in β-lactam hypersusceptibility. Thus, we did not observe excess secretion of PrsA in NaHCO_3_-responsive strains that could explain the decreased PrsA levels within the CM. These results suggest that NaHCO_3_ is not directly affecting PrsA functionality but rather more directly affecting overall PrsA protein levels. NaHCO_3_ reduces the expression of both *vraSR* and *prsA*, thereby decreasing the overall production and CM localization of the PrsA chaperone, potentially impairing the ultimate maturation and functionality of PBP2a within the CM.

Third, NaHCO_3_ reduced *pbp4* expression selectively in NaHCO_3_-responsive strains. Importantly, *pbp4* is a nonessential PBP that functions as an auxiliary transpeptidase during cell wall synthesis (32). Of relevance to the present study, deletion of *pbp4* in certain community-acquired MRSA strains (e.g., USA300 and MW2, both NaHCO_3_ responsive) resulted in a 16-fold increased susceptibility to oxacillin, while this deletion had no effect on oxacillin MICs in strain COL (NaHCO_3_ nonresponsive) (26). Further, in USA300 and MW2, *pbp4* mutants exposed to oxacillin exhibited substantially less highly cross-linked cell wall peptidoglycan, indicating a cooperative action between PBP2a and PBP4 in cell wall synthesis during β-lactam exposure. As demonstrated by our data, NaHCO_3_ reduces both the transcription of *pbp4* and overall production of PBP2a, which likely leads to a net decrease in highly cross-linked peptidoglycan in NaHCO_3_-responsive strains. Although little is known about the regulation of *pbp4* expression (33), deletion of *pbp4* results in a decrease in *pbp2* transcription (26). Recent analyses of the transcriptomes of NaHCO_3_-responsive strains by RNA sequencing from our laboratory confirm that NaHCO_3_ downregulates *pbp2* expression (unpublished data); this suggests that NaHCO_3_ is acting on an upstream regulator of both *pbp2* and *pbp4*. Interestingly, PBP2 is one of only three known enzymes with transglycosylase activity in S. aureus (34), indicating that NaHCO_3_ has additional impacts on peptidoglycan transglycosylation in responsive MRSA strains. Further work is needed to understand the specific impact of NaHCO_3_ on the regulation of the overall cadre of genes involved in peptidoglycan biosynthesis as well as the impact of NaHCO_3_ on the abundance of highly cross-linked peptidoglycan muropeptides within the MRSA cell wall. These investigations are ongoing in our laboratories.

Lastly, in addition to strain-specific and selective impacts described above in NaHCO_3_-responsive versus -nonresponsive MRSA, NaHCO_3_ also had several, more global effects in all eight prototype strains that could impact CM physiology. For example, NaHCO_3_ reduced the activity of the global stress response regulator, SigB (35, 36), in all strains studied. In S. aureus, SigB upregulates the expression of the operon *crtOPQMN*, which is responsible for the biosynthesis of the triterpenoid carotenoid pigment staphyloxanthin (17, 37). Staphyloxanthin, in turn, is a crucial factor in the formation of FMMs, which create the scaffolding for oligomerization of PBP2a within the CM (19). Interestingly, disruption of staphyloxanthin synthesis by clinical statin agents in S. aureus leads to increased susceptibility to β-lactams (19). As expected, based on the NaHCO_3_ repression of SigB activity, carotenoid production was concomitantly reduced in all strains tested. Conversely, NaHCO_3_ exposure resulted in increased *floA* expression in all strains tested, highlighting the possibility that NaHCO_3_ is disrupting the normal equilibrium of two key factors necessary for stable FMM formation (i.e., relative proportional contents of carotenoids and FloA). This disequilibrium could contribute to the NaHCO_3_-responsive phenotype when other observed alterations in PBP2a maturation are also present.

There are several limitations to our investigations. For example, we only studied a relatively small number of MRSA strains (*n* = 8). Additionally, we carefully selected only a specific cadre of genes to study that have been well characterized as to their known impacts on PBP2a regulation, production, and functionality. Further, we have not yet investigated how changes in gene expression impact cell wall morphology and FMM composition/organization. Moreover, the more global genetic impacts of NaHCO_3_ in MRSA await more extensive analyses, such as whole-genome sequencing and RNA sequencing, studies that are in progress. Finally, genetic swapping studies between NaHCO_3_-responsive and -nonresponsive strains will be required to more precisely define the role of specific PBP2a genotype species in the β-lactam sensitization phenomenon related to NaHCO_3_ exposures (38). Importantly, understanding the mechanism(s) of NaHCO_3_ responsiveness may help guide future clinical microbiologic laboratory screening strategies as well as the repurposing of treatment practices for MRSA infections, resulting in improved patient outcomes using β-lactam-based therapies.

**FIG 6:**
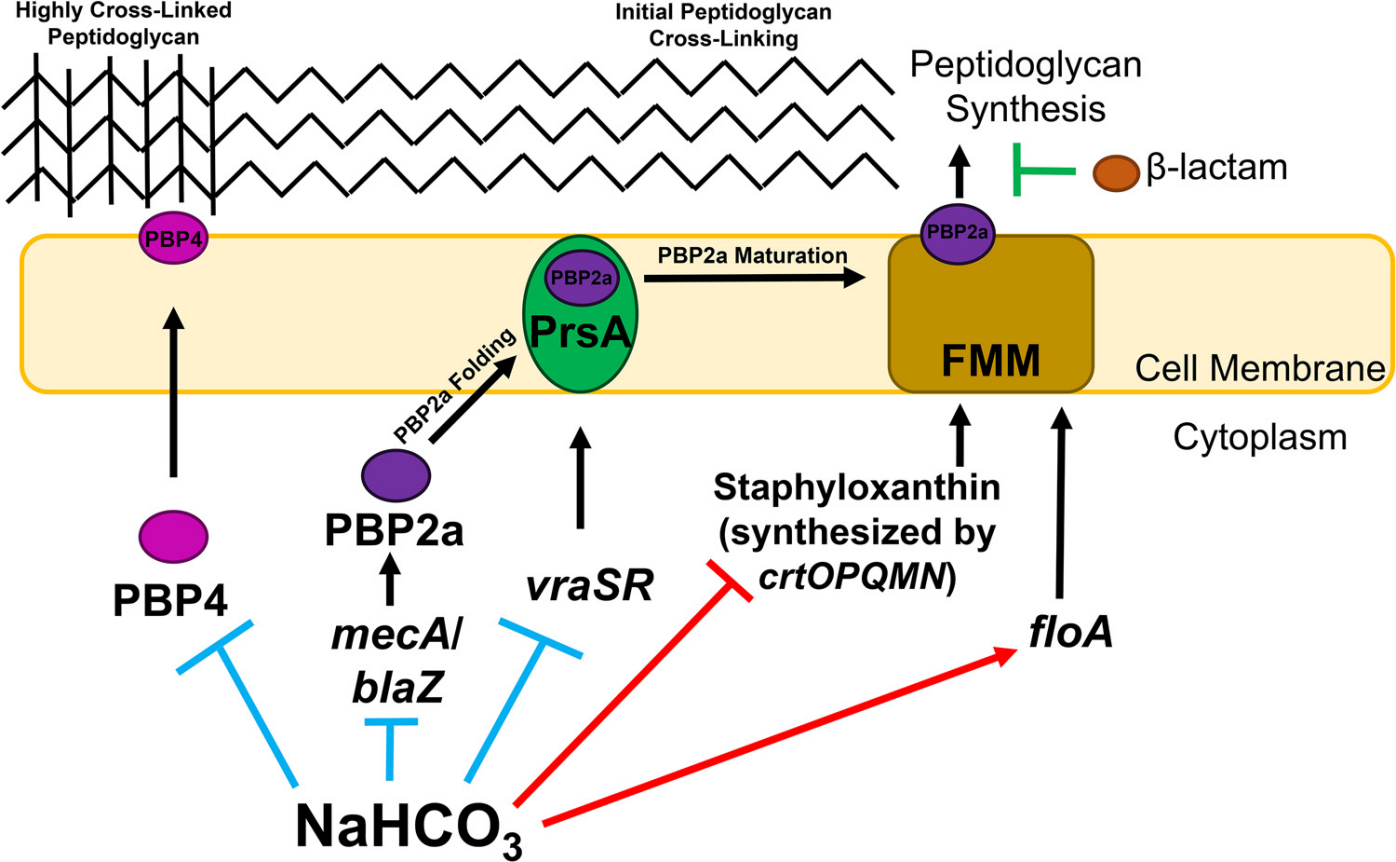
Schematic overview of the impact of NaHCO_3_ on PBP2a maturation/function and peptidoglycan biosynthesis. Impacts specific to NaHCO_3_-responsive strains (blue inhibition symbols) include the repression of PBP2a protein production, *pbp4* gene expression, *vraSR* gene expression, and PrsA protein production. Impacts of NaHCO_3_ on all strains tested include repression of carotenoid production (red inhibition symbol) and increased *floA* gene expression (red enhancement symbol), potentially resulting in less stable functional membrane microdomains (FMMs). Anticipated collateral impact on NaHCO_3_-responsive strains is an overall decrease in PBP2a mediated peptidoglycan synthesis and the generation of highly cross-linked peptidoglycan, resulting in increased susceptibility to β-lactam antibiotics.

Figure 6 represents one proposed pathway for the overall impact of NaHCO_3_ on key MRSA genotypes and phenotypes, based on our current investigations.

In summary, NaHCO_3_ was found to have profound effects on PBP2a that were specific to NaHCO_3_-responsive strains (e.g., *mecA* and *blaZ* expression, PBP2a protein production and CM localization, and expression of genes involved in PBP2a maturation and functionality). In contrast, NaHCO_3_ impacted certain genotypes-phenotypes more globally in both NaHCO_3_-responsive and -nonresponsive MRSA (i.e., SigB activity, downstream carotenoid production, and *floA* expression). The latter data indicate that certain NaHCO_3_-mediated impacts are themselves involved and necessary in the collective NaHCO_3_-responsive outcome in MRSA, but they are not individually sufficient to cause this overall phenotype without additional perturbations in PBP2a production, maturation, and functionality (Fig. 6).

## MATERIALS AND METHODS

### Bacterial strains and media.

All MRSA strains used in this study were initially isolated from patients with invasive clinical infections: MRSA 11/11, MW2, PB 043-043, C24, COL, BMC1001, PB 027-133, and PB 017-037 (12, 22, 39–41). Four of these strains (MRSA 11/11, MW2, BMC1001, and COL) were selected based on their inclusion in a prior study on the NaHCO_3_-responsive phenomenon (11). The other four strains (PB 043-043, C24, PB 027-133, and PB 017-037) were selected from a larger screen of NaHCO_3_-responsive MRSA (12) based on their oxacillin and cefazolin MICs in the presence/absence of NaHCO_3_ and their clonal complex (CC) types. The CC types of 7/8 isolates represent genotypes in current widespread clinical circulation (i.e., CC types 5 and 8) (see Table S1 in the supplemental material) (42). MRSA 11/11, MW2, PB 043-043, C24, BMC1001, PB 027-133, and PB 017-037 are all β-lactamase positive; COL is naturally β-lactamase negative. MICs were determined, in the presence or absence of NaHCO_3_, by standard CLSI methods (Table S1). MICs for MRSA 11/11, MW2, COL, and BMC1001 have been previously reported (11).

For all experiments, strains were grown in ambient air at 37°C in cation-adjusted Mueller-Hinton Broth (CA-MHB; Difco) plus 100 mM Tris (to stabilize the pH at ∼7.3 ± 0.1) with or without 44 mM NaHCO_3_ (the optimal concentration to disclose the NaHCO_3_-responsive phenotype *in vitro* and *ex vivo*, reflective of deep-tissue HCO_3_ levels [11–13, 43]). For oxacillin exposure experiments, 0.5× MIC oxacillin was used for a given strain in the specified growth medium, with 2% NaCl incorporated into testing media (Table S1).

### MIC assays.

The MICs of oxacillin were determined by broth microdilution according to CLSI guidelines in specified media, as previously described (9, 11, 44). All MICs are the mode of at least six independent determinations.

### PBP2a latex agglutination assays.

A semiquantitative, rapid, and reliable latex agglutination method (Seiken, Tokyo, Japan) was used to approximate total PBP2a production, as previously described (11). Agglutination results were scored as high (+++), moderate (++), low (+), or negative (−) based on the presence or absence of an overt agglutination pattern, corresponding to the total amount of PBP2a in the sample. S. aureus ATCC 43300 (MRSA; PBP2a positive) and ATCC 25923 (MSSA; PBP2a negative) were used as positive and negative controls, respectively, in all assays. The grading of agglutination assays was performed blindly as to strain identifications by one of the investigators (S. C. Ersoy).

### Membrane (CM) protein extraction.

Cells were grown to stationary phase (PBP2a) or log phase (optical density at 600 nm [OD_600_], 0.5; for PrsA) in CA-MHB plus 100 mM Tris plus 0.5× MIC oxacillin, with or without 44 mM NaHCO_3_ (Table S1), and their centrifuged pellets were then resuspended in phosphate-buffered saline (PBS), pH 7.3. Thereafter, 1 ml of resuspended pellets was incubated with 10 μl each of DNase (Ambion, Invitrogen), RNase (Thermo Fisher Scientific), and Halt protease inhibitor cocktail (Thermo Fisher Scientific) for 30 min at 37°C. Cells were then mixed with glass beads and disrupted using a FastPrep cell disrupter. Disrupted cells were centrifuged for 20 min at 4°C and 13,000 rpm, and the supernatant was ultracentrifuged for 2 h at 4°C and 15,000 rpm to collect total CM proteins. The CM protein pellets were resuspended in PBS, and protein concentrations were quantified by Bradford protein assay. Isolated protein extracts were stored at −80°C.

### Western blotting for PBP2a and PrsA protein expression.

Forty micrograms of CM protein extract was separated on a 4 to 12% Bis-Tris gel (Invitrogen) and then blotted onto a nitrocellulose membrane (Amersham). Total protein loading was confirmed by staining with 0.25% Ponceau reagent (Fig. S1). The membrane was blocked with 10% dry milk in Tris-buffered saline with Tween (TBST). PBP2a was probed with a chicken anti-PBP2a antibody (RayBiotech) diluted 1:2,500 and detected with an anti-chicken IgY cross-absorbed secondary antibody, horseradish peroxidase (Thermo Fisher Scientific), diluted 1:5,000. In parallel studies, PrsA was probed with a chicken anti-PrsA antibody diluted 1:2,500 (21). Labeled proteins were imaged using a c400 imager (Azure Biosystems). Western blotting for PrsA in strain BMC1001 could not be performed due to a single-nucleotide deletion within its *prsA* coding region, causing a frameshift mutation resulting in a premature stop codon (*prsA* g. 796_798del; p. L272X).

### Isolation of RNA and qRT-PCR analysis.

RNA was isolated from strains as detailed previously (11). Quantitative real-time PCR (qRT-PCR) was performed using primers for *blaZ*, *prsA*, *vrsASR*, *pbp4*, *floA*, *mecA*, and *gyrB* as previously described (15, 25, 45–47); these primers are listed in Table S2. The *gyrB* gene was used as a housekeeping gene for transcript normalization (verification of the adequacy of *gyrB* as a reproducible control for these assays was independently done using *rpoB* in parallel experiments [data not shown]) (48). Relative gene expression was calculated using the 2^−ΔΔCT^ method from two independent biological replicates performed in triplicate on at least 2 separate runs for each strain/condition. Data are presented as the relative gene expression in CA-MHB plus 100 mM Tris plus 44 mM NaHCO_3_ normalized to the relative gene expression in CA-MHB plus 100 mM Tris for each strain, where expression in CA-MHB 100 mM Tris was set to 1.0. A Student’s *t* test was used for all statistical analyses.

### Carotenoid production assay.

To quantify relative levels of staphyloxanthin production, carotenoids were isolated by methanol extraction as previously described (37). Briefly, cells were grown overnight in specified medium, pelleted, and washed twice with PBS. Methanol was added to pellets at a volume that was normalized to total cell weight for each sample (0.0625 g cell weight/ml of methanol). Samples were vortexed vigorously for 10 min to allow carotenoid extraction from the cell pellet into methanol. Methanol absorbance was measured at the OD_450_. A higher absorbance reading indicates greater carotenoid production. The absorbance for cells grown in CA-MHB plus 100 mM Tris plus 44 mM NaHCO_3_ was normalized to the absorbance in CA-MHB plus 100 mM Tris for each strain, where CA-MHB plus 100 mM Tris equals 1.0. A Student’s *t* test was used for all statistical analyses.

### Data availability.

The whole-genome shotgun project for strains BMC1001, COL, MRSA 11/11, and MW2 have been deposited at DDBJ/ENA/GenBank as a BioProject (PRJNA697971) under the accession numbers JAFFHU000000000, JAFFHV000000000, JAFFHW000000000, and JAFFHX000000000. The versions described in this paper are versions JAFFHU010000000, JAFFHV010000000, JAFFHW010000000, and JAFFHX010000000.

## Supplementary Material

Supplemental file 1
