# Supplementary material for: Impact of Bicarbonate on PBP2a Production, Maturation, and Functionality in Methicillin-Resistant Staphylococcus aureus
Source: Antimicrob Agents Chemother. 2021 Apr 19;65(5):e02621-20. doi: 10.1128/AAC.02621-20 (PMC8092911; doi:10.1128/AAC.02621-20)
Supplement: Supplemental file 1 [file AAC.02621-20-s0001.pdf]

Figure S1

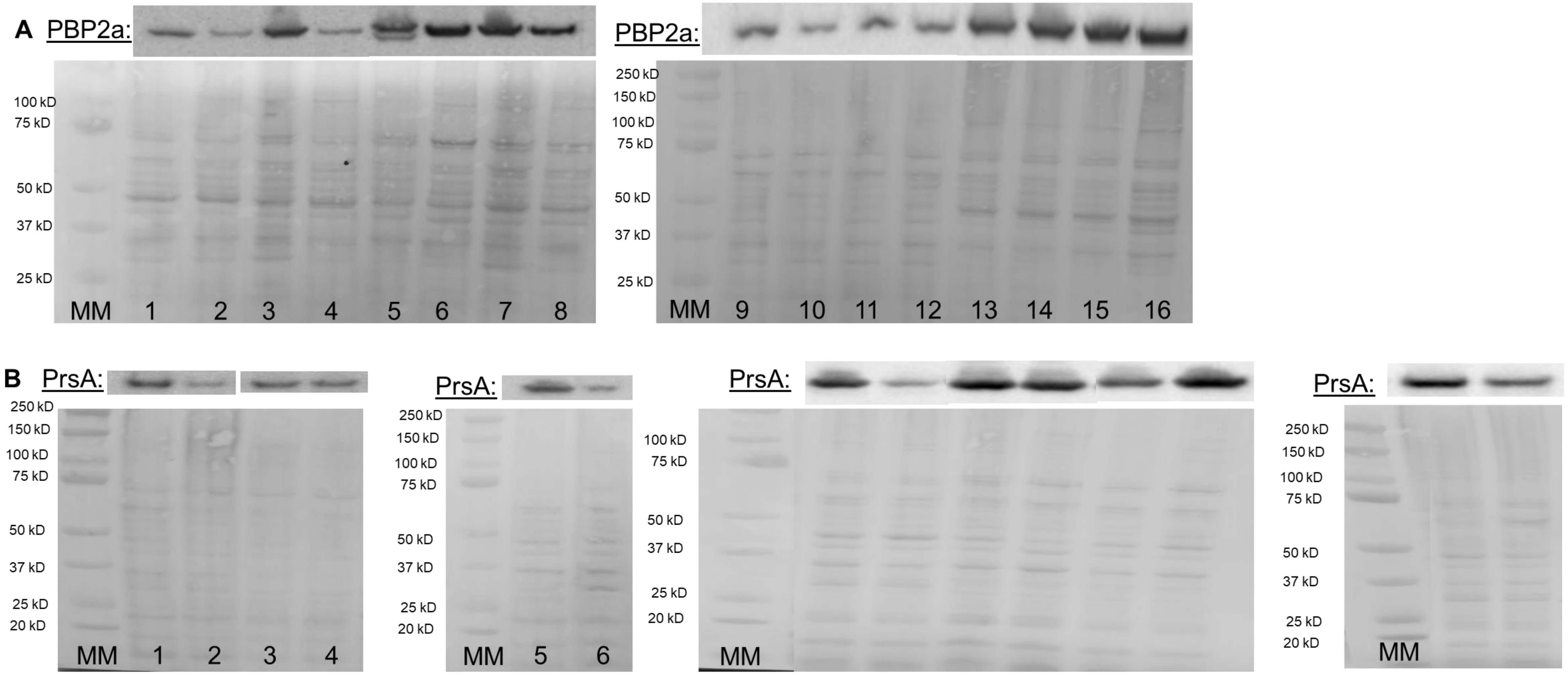

A
PBP2a:
100 kD
75 kD
50 kD
37 kD
25 kD
MM 1 2 3 4 5 6 7 8
PBP2a:
250 kD
150 kD
100 kD
75 kD
50 kD
37 kD
25 kD
MM 9 10 11 12 13 14 15 16
B
PrsA:
250 kD
150 kD
100 kD
75 kD
50 kD
37 kD
25 kD
20 kD
MM 1 2 3 4
PrsA:
250 kD
150 kD
100 kD
75 kD
50 kD
37 kD
25 kD
20 kD
MM 5 6
PrsA:
100 kD
75 kD
50 kD
37 kD
25 kD
20 kD
MM
PrsA:
250 kD
150 kD
100 kD
75 kD
50 kD
37 kD
25 kD
20 kD
MM

Figure S1. Total protein stain from PBP2a (**A**) and PrsA (**B**) Western blots. Cell membrane proteins were extracted from stationary phase cells. Lane numbers correspond to the following samples in panel A: (1) MRSA 11/11 w/o (2) MRSA 11/11 + $NaHCO_3$ (3) MW2 w/o (4) MW2 + $NaHCO_3$ (5) COL w/o (6) COL + $NaHCO_3$ (7) BMC1001 w/o (8) BMC1001 + $NaHCO_3$ (9) PB 043-043 w/o (10) PB 043-043 + $NaHCO_3$ (11) C24 w/o (12) C24 + $NaHCO_3$ (13) PB 027-133 w/o (14) PB 027-133 + $NaHCO_3$ (15) PB 017-037 w/o (16) PB 017-037 + $NaHCO_3$. Lane numbers correspond to the following samples in panel B: (1) MRSA 11/11 w/o (2) MRSA 11/11 + $NaHCO_3$ (3) COL w/o (4) COL + $NaHCO_3$ (5) MW2 w/o (6) MW2 + $NaHCO_3$ (7) C24 w/o (8) C24 + $NaHCO_3$ (9) PB 027-133 w/o (10) PB 027-133 + $NaHCO_3$ (11) PB 017-037 w/o (12) PB 017-037 + $NaHCO_3$ (13) PB 043-043 w/o (14) PB 043-043 + $NaHCO_3$

Figure S2

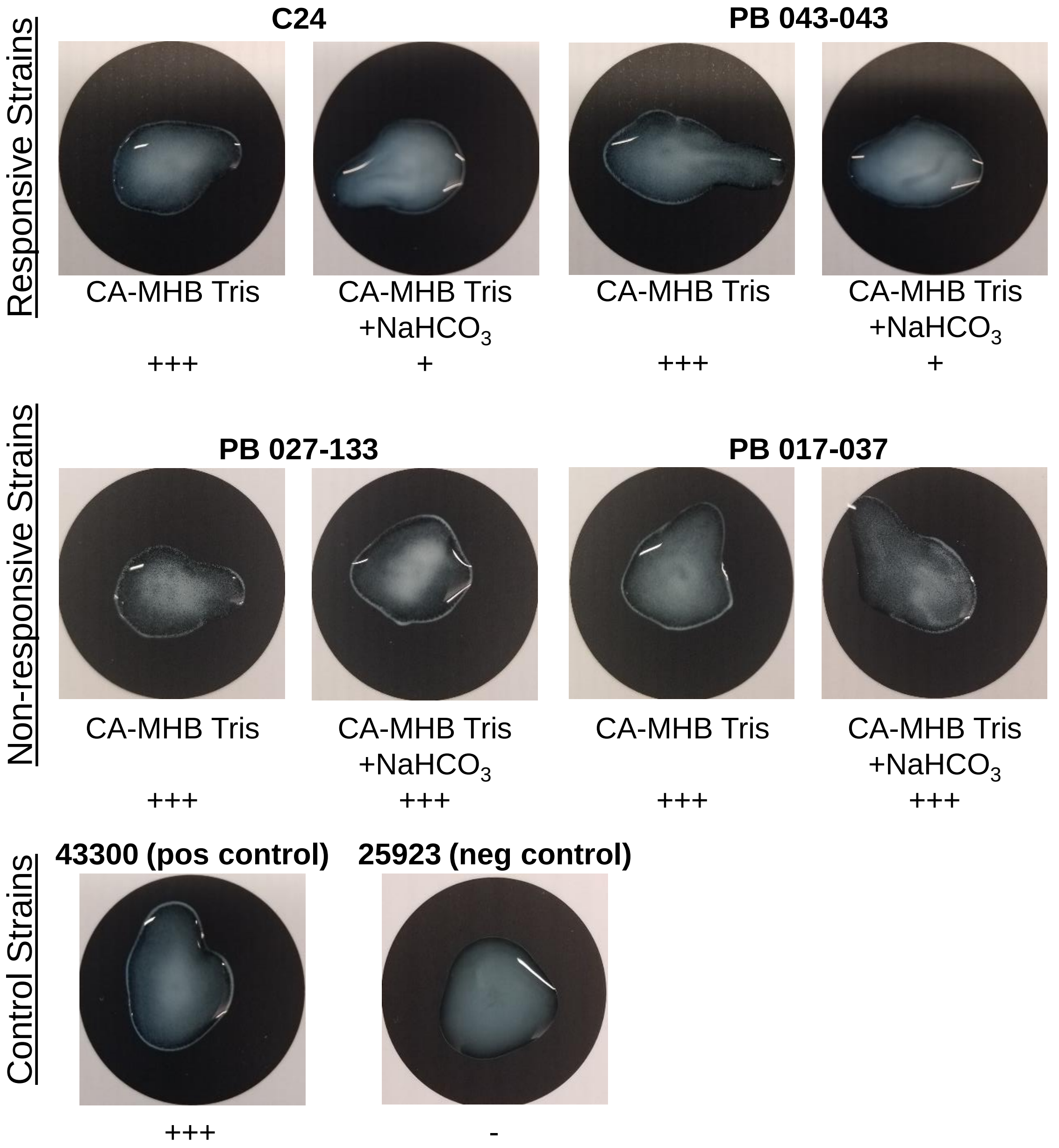

Figure S2. PBP2a agglutination of responsive (PB 043-043; C24) and non-responsive strains (PB 027-133; PB 017-037). Strains ATCC 43300 and ATCC 25923 were used as positive and negative controls, respectively.

Table S1. Oxacillin minimum inhibitory concentrations (MICs, mg/L) and cloncal complex (CC) types of strains used in this study. In experiments in which sub-MIC concentrations of oxacillin were used, cells were exposed to 1/2 of the concentrations indicated in this table for each strain/condition.

Strain	Ca-MHB 100 mM Tris	Ca-MHB 100 mM Tris 44 mM $NaHCO_3$	CC Type
MRSA 11/11	32	0.5	8
MW2	64	2	1
PB 043-043	16	1	8
C24	32	1	8
COL	512	512	8
BMC1001	256	256	8
PB 027-133	256	256	5
PB 017-037	32	32	5

Table S2. Primers used in this study for qRT-PCR gene expression analyses.

Gene Name	Primer Sequences	Reference
mecA	Fwd 5' TCCAGATTACAACTTCACCAGG 3'<br>Rev 5' CCACTTCATATCTTGTAACG 3'	1
blaZ	Fwd 5’ CTTTAAAAGAACTTATTGAGGCTTCA 3'<br>Rev 5’ CCACCGATTTCTTTTATAATTT 3’	This Study
prsA	Fwd 5' AGTTAATGATAAGAAGATTGACGA 3'<br>Rev 5' GAAGGGCCTTTTCAAATTTATCTTT 3'	2
vraSR	Fwd 5’ AATGGAAGGCGAAACAGTTG 3’<br>Rev 5’ TCGCAACTTATGCAATTCTTG 3’	This Study
pbp4	Fwd 5' TGGTGCTAACTGCTTTGTAA 3'<br>Rev 5' GCTAAAGCTATCGGAATGAA 3'	3
floA	Fwd 5' CTAGTCCTGCTTTGTGCGCT 3'<br>Rev 5' TTAGCAGCTGGCGTTCATGT 3'	4
gyrB	Fwd 5' CGCAGGCGATTTTACCATTA 3'<br>Rev 5' GCTTTCGCTAGATCAAAGTCG 3'	5
rpoB	Fwd 5' CTA AGC ACA GAG GTC GT 3'<br>Rev 5' ACG GCA TCC TCA TAG T 3'	6

SUPPLEMENTAL REFERENCES

1. Li L, Cheung A, Bayer AS, Chen L, Abdelhady W, Kreiswirth BN, Yeaman MR, Xiong YQ. 2016. The global regulon *sarA* regulates beta-Lactam antibiotic resistance in methicillin-resistant *Staphylococcus aureus in vitro* and in endovascular infections. J Infect Dis 214:1421-1429.

2. Jousselin A, Manzano C, Biette A, Reed P, Pinho M, Rosato A, Kelley WL, Renzoni A. 2015. The *Staphylococcus aureus* chaperone PrsA is a new auxiliary factor of oxacillin resistance affecting penicillin-binding protein 2A. Antimicrob Agents Chemother:AAC. 02333-15.

3. Dumitrescu O, Choudhury P, Boisset S, Badiou C, Bes M, Benito Y, Wolz C, Vandenesch F, Etienne J, Cheung AL. 2011. β-lactams interfering with PBP1 induce panton-valentine leukocidin expression by triggering *sarA* and *rot* global regulators of *Staphylococcus aureus*. Antimicrob Agents Chemother 55:3261-3271.

4. Mielich-Süss B, Wagner RM, Mietrach N, Hertlein T, Marincola G, Ohlsen K, Geibel S, Lopez D. 2017. Flotillin scaffold activity contributes to type VII secretion system assembly in *Staphylococcus aureus*. PLoS Pathog 13:e1006728.

5. Seidl K, Chen L, Bayer AS, Hady WA, Kreiswirth BN, Xiong YQ. 2011. Relationship of *agr* expression and function with virulence and vancomycin treatment outcomes in experimental endocarditis due to methicillin-resistant *Staphylococcus aureus*. Antimicrob Agents Chemother 55:5631-5639.

6. Sihto H-M, Tasara T, Stephan R, Johler S. 2014. Validation of reference genes for normalization of qPCR mRNA expression levels in *Staphylococcus aureus* exposed to osmotic and lactic acid stress conditions encountered during food production and preservation. FEMS Microbiol Lett 356:134-140.
